# Long Noncoding RNAs Promote Transcriptional Poising of Inducible Genes

**DOI:** 10.1371/journal.pbio.1001715

**Published:** 2013-11-19

**Authors:** Sara C. Cloutier, Siwen Wang, Wai Kit Ma, Christopher J. Petell, Elizabeth J. Tran

**Affiliations:** 1 Department of Biochemistry, Purdue University, West Lafayette, Indiana, United States of America; 2 Purdue University Center for Cancer Research, Purdue University, West Lafayette, Indiana, United States of America; Harvard Medical School, United States of America

## Abstract

Long noncoding RNAs (lncRNAs) are a class of molecules that impinge on the expression of protein-coding genes. Previous studies have suggested that the *GAL* cluster-associated lncRNAs of *Saccharomyces cerevisiae* repress expression of the protein-coding *GAL* genes. Herein, we demonstrate a previously unrecognized role for the *GAL* lncRNAs in activating gene expression. In yeast strains lacking the RNA helicase, *DBP2*, or the RNA decay enzyme, *XRN1*, we find that the *GAL* lncRNAs specifically accelerate gene expression from a prior repressive state. Furthermore, we provide evidence that the previously suggested repressive role is a result of specific mutant phenotypes, rather than a reflection of the normal, wild-type function of these noncoding RNAs. To shed light on the mechanism for lncRNA-dependent gene activation, we show that rapid induction of the protein-coding *GAL* genes is associated with faster recruitment of RNA polymerase II and reduced association of transcriptional repressors with *GAL* gene promoters. This suggests that the *GAL* lncRNAs enhance expression by derepressing the *GAL* genes. Consistently, the *GAL* lncRNAs enhance the kinetics of transcriptional induction, promoting faster expression of the protein-coding *GAL* genes upon the switch in carbon source. We suggest that the *GAL* lncRNAs poise inducible genes for rapid activation, enabling cells to more effectively trigger new transcriptional programs in response to cellular cues.

## Abstract 2

The GAL cluster-associated long non-coding RNAs (lncRNAs) promote rapid induction of GAL genes in budding yeast, thereby promoting a faster switch in transcriptional programs when needed.

## Author Summary

Long noncoding RNAs (lncRNAs) are a recently identified class of molecules that regulate the expression of protein-coding genes through a number of mechanisms, some of them poorly characterized. The *GAL* gene cluster of the yeast *Saccharomyces cerevisiae* encodes a series of three inducible genes that are turned on or off by the presence or absence of specific carbon sources in the environment. Previous studies have documented the presence of two lncRNAs—*GAL10* and *GAL10s*—encoded by genes that overlap the *GAL* cluster. We have now uncovered a role for both these lncRNAs in promoting the activation of the *GAL* genes when they are released from repressive conditions. This activation occurs at the kinetic level, through more rapid recruitment of RNA polymerase II and decreased association of the co-repressor, Cyc8. Under normal conditions, but also especially when they are stabilized and their levels are up-regulated, these *GAL* lncRNAs promote faster *GAL* gene activation. We suggest that these lncRNA molecules poise inducible genes for quick response to extracellular cues, triggering a faster switch in transcriptional programs.

## Introduction

Essential cellular processes, such as growth, organ development, and differentiation, require precise spatial and temporal control of gene expression. Eukaryotes have developed intricate pathways for regulating gene expression at the transcriptional level in both global and gene-specific manners [1],[2]. Recent studies have provided evidence that lncRNA molecules facilitate transcriptional control of protein-coding genes [3],[4]. Thus far, the most well-characterized lncRNA is *Xist*, which facilitates X chromosome inactivation in mammalian cells [5],[6]. Similar to a transcription factor, *Xist* functions by directing corepressor complexes to the targeted DNA loci [7]. Other examples of repressive lncRNAs include *HOTAIR*, a 2.1 kilobase transcript that directs repression of developmental gene loci, and *PANDA*, which regulates cell-cycle–dependent gene expression [8],[9]. Recruitment of transcription factors may also be a primary mechanism for lncRNAs associated with transcriptional activation [10]–[13], suggesting that these molecules may recruit both activators and repressors. Other lncRNAs, however, appear to function solely through their synthesis, whereby the act of transcription alters the chromatin structure of a targeted gene promoter [14]–[16]. This diversity of action may account for the fact that individual lncRNAs are more conserved in their position than in their nucleotide sequence [17]. Interestingly, many mammalian lncRNAs are associated with genes that require precise temporal control of initiation to facilitate proper cell growth and differentiation [9],[13],[18]–[23]. This suggests that these molecules may control the timing of gene expression in response to specific signals.

The *GAL10* lncRNA in the *S. cerevisiae* budding yeast model system is encoded within the *GAL* gene cluster, which is composed of the *GAL1*, *GAL10*, and *GAL7* metabolic genes required for utilization of galactose as a carbon source [24]–[26]. Budding yeast are able to utilize glucose and this catabolite is the preferential carbon source for energy production. However, yeast also has the capacity to utilize galactose when it is the sole carbon source in the media [27],[28]. The transition from glucose to galactose metabolism requires an intricate switch in transcriptional programs, whereby genes repressed in the presence of glucose must be activated for production of galactose metabolizing enzymes [29]–[31]. The highly orchestrated series of events required to facilitate this *GAL* gene metabolic switch is well understood and involves modulation of carbon source-dependent transcriptional activators and repressors [1], [29], [32]–[34]. Interestingly, the *GAL10* lncRNA has been proposed to act additively with transcriptional repressors, to provide tighter control of this gene expression network [24], [25], [35], [36]. The repressive role of the *GAL10* lncRNA is supported by correlative changes in histone acetylation patterns and the observation that impaired lncRNA degradation in RNA decay-deficient mutants results in defective expression of the *GAL1* and *GAL10* genes [24], [25]. However, the mechanism for repression has not been established, and unlike *Xist*, there is no evidence for a direct interaction between the *GAL10* lncRNA and a transcriptional repressor.

Our laboratory recently found that loss of the RNA helicase *DBP2* results in up-regulation of another lncRNA within the *GAL* cluster, termed *GAL10s* [37], [38]. To determine the role of Dbp2 in this process, we initially set out to test the hypothesis that the *GAL10s* lncRNA also functions in transcriptional repression, similar to the *GAL10* lncRNA. Surprisingly, this revealed an unexpected and uncharacterized role for both of the *GAL* lncRNAs in promoting gene activation. We suggest that these findings identify a novel role for the *GAL* lncRNAs in poising protein-coding genes for rapid induction in response to cellular and environmental cues.

## Results

### The *GAL7* and *GAL10* Genes Are Rapidly Induced from Repressed Conditions in *dbp2Δ* Cells as Compared to Wild Type

**Figure 1:**
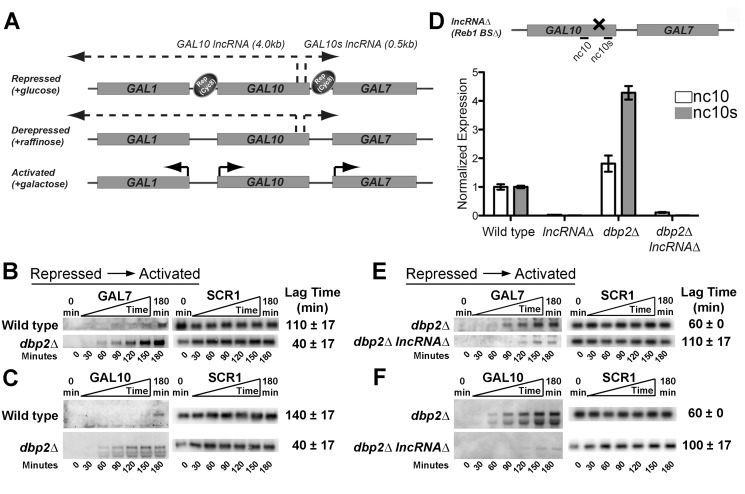
Loss of *DBP2* results in rapid, lncRNA-dependent induction of *GAL10* and *GAL7* from repressed conditions. (A) Simplified model for carbon-source-dependent regulation of *GAL1*, *GAL7*, and *GAL10* genes within the *GAL* cluster. Glucose-dependent repression is mediated by transcription factors (not shown), which then recruit other proteins such as the Tup1–Cyc8 co-repressor complex to promote repression [28], [32], [40], [46], [47], [51]. Derepression occurs under nonrepressing, noninducing conditions when the repressors are no longer present and the *GAL* genes are not transcriptionally active [29]. Activation only occurs in the presence of galactose [1], [29]. Synthesis of the *GAL10* lncRNA, and likely the *GAL10s* lncRNA, is mutually exclusive with activated expression of the *GAL* genes [24], [25]. (B–C) *GAL7* (B) and *GAL10* (C) genes are rapidly induced in *dbp2Δ* cells following a switch from repressed to activated conditions. Transcriptional induction of wild-type (BY4741) and *dbp2Δ* strains was conducted by isolating RNA from cells at 30 min intervals prior to and immediately following a nutritional shift from repressive (+glucose) to activated (+galactose) conditions. Transcripts were detected by northern blotting using ^32^P-labeled, double-stranded (ds)DNA probes corresponding to *GAL7*, *GAL10*, or *SCR1* RNA as indicated. Each time course was conducted in triplicate. Average lag times to induction are shown with the standard deviation (s.d.) for three, independent biological replicates and correspond to the first time point in a series of time points with increasing *GAL* transcript levels after normalization to *SCR1*. An s.d. of zero indicates no variation between biological samples with 30 min time points, whereas an s.d. of 17 indicates a variance of 30 min between replicates. (D, Top) Schematic diagram of the *lncRNAΔ* strain with *GAL10* and *GAL10s* lncRNAs and primer sets for RT-qPCR. The four previously identified binding sites for the Reb1 transcription factor are present within the 3′ end of the *GAL10* coding region [24]. The *lncRNA*Δ harbors silent mutations that disrupt all binding sites for the Reb1 transcription factor [24]. (D, Bottom) The *lncRNA*Δ mutation abolishes expression of both the *GAL10* and *GAL10s* lncRNA in wild-type and *dbp2*Δ cells. *GAL10* and *GAL10s* lncRNAs were detected in the indicated strains following growth in glucose using RT-qPCR as previously described with primers nc10 and nc10s [37]. Transcript levels were normalized to *ACT1*, which does not vary between these strains, and is the average of three biological replicates with respect to wild type and standard error from the mean (SEM). (E–F) Loss of *GAL10* and *GAL10s* lncRNAs restores repression at *GAL7* (E) and *GAL10* (F) loci in *DBP2*-deficient cells. Transcriptional induction assays from repressive conditions were conducted with isogenic *dbp2*Δ and *dbp2*Δ* lncRNA*Δ strains as in Figure 1B–C.

The *GAL* cluster is a group of gene loci that have been extensively utilized to define the mechanism and order of events in transcriptional regulation [1], [27]–[29], [39]. The cluster encodes three genes, *GAL1*, *GAL7*, and *GAL10*, which exist in three distinct transcriptional states in response to carbon sources: repressed (+glucose), derepressed (+raffinose), and activated (+galactose) (Figure 1A). This cluster also encodes the *GAL10* lncRNA, which is a 4.0 kb antisense transcript that overlaps *GAL10* and *GAL1*, and the *GAL10s* lncRNA, a 0.5 kb sense-oriented transcript upstream of *GAL7* (Figure 1A) [24],[26], [38]. The protein-coding *GAL* genes are regulated by carbon source-responsive repressors and activators (Figure 1A) [27], [29], [32]. In contrast, the *GAL* lncRNAs are expressed when the protein-coding *GAL* genes are transcriptionally inactive (+glucose or raffinose) [24]–[26] and are dependent on the transcription factor, Reb1 (Figure 1D) [24], [26].

Previous studies from our laboratory demonstrated that loss of the RNA helicase *DBP2* results in accumulation of a 3′ extended *GAL10s* lncRNA under conditions when the protein-coding *GAL* genes are transcriptionally repressed (+glucose) [37]. Based on previous evidence that up-regulation of the *GAL10* lncRNA impairs transcriptional activation of the *GAL1* and *GAL10* genes [25], we anticipated that loss of *DBP2* would similarly delay transcriptional activation of *GAL7*. To this end, we analyzed the transcriptional induction profile of *GAL7* in wild-type and *dbp2Δ* cells following a media shift from repressed to activated conditions (glucose to galactose) by isolating RNA fractions over time at 30 min intervals from three, independent biological replicates per strain. We then conducted northern blotting of isolated RNAs and then obtained a semiquantitative estimate of the degree of repression by calculating the average lag time or time to the first appearance of *GAL7* transcripts after normalization to the *SCR1* loading control (Figure 1B). In contrast to wild-type cells, which exhibited a normal, ∼2-h lag time to induction [40], [41], *dbp2Δ* cells displayed detectible *GAL7* transcripts within an average of 40 min (Figure 1B). This was unexpected and suggested that loss of *DBP2* results in a rapid induction of *GAL7* expression from repressive conditions. To determine if the requirement for *DBP2* is specific to *GAL7*, we then assayed *GAL10* induction (Figure 1B, bottom). This revealed that *GAL10* is also rapidly induced in *dbp2*Δ cells (Figure 1C). In addition to full-length *GAL10* transcripts, we also observed the appearance of shorter *GAL10* products, which are likely the result of previously noted cryptic initiation defects in *dbp2*Δ cells (Figure 1C, bottom) [37]. Regardless, this reveals that the loss of *DBP2* results in rapid induction of both the *GAL7* and *GAL10* genes from repressed (+glucose) conditions.

### Loss of the *GAL* lncRNAs Restores Repression in *dbp2Δ* Cells

The results above suggest that *DBP2* is required to maintain glucose-dependent repression of the protein-coding *GAL* genes. To determine if this requirement is dependent on the presence of the *GAL* lncRNAs, we constructed a *dbp2*Δ* lncRNA*Δ strain that lacks expression of both of the *GAL10* and *GAL10s* lncRNA molecules. Expression of the *GAL10* lncRNA is dependent on the Reb1 transcription factor, which has four putative binding sites within the 3′ end of the *GAL10* coding region [24], [26]. Although it is not known which Reb1 site(s) is necessary for expression of the *GAL10* lncRNA, previous studies have shown that the *lncRNA*Δ strain, which harbors silent mutations of all four sites, abolishes synthesis of this lncRNA (Figure 1D) [24]. Because the *GAL10* and *GAL10s* lncRNAs arise from juxtaposed sites within the protein-coding *GAL10* gene, we speculated that the *lncRNA*Δ mutation would also abolish synthesis of the *GAL10s* lncRNA. To test this, we conducted reverse transcription-quantitative PCR (RT-qPCR) analysis to measure lncRNA abundance in isogenic wild-type, *dbp2*Δ, *lncRNA*Δ, and *dbp2*Δ* lncRNA*Δ cells grown in the presence of glucose, using primers positioned within the 5′ ends of the respective lncRNAs (nc10 and nc10s in Figure 1D). This revealed a slight increase in the *GAL10* lncRNA and greater overabundance of the *GAL10s* lncRNA in the *dbp2*Δ strain similar to previous studies [37]. More importantly, neither the *GAL10* nor the *GAL10s* lncRNA were detectible in strains harboring the *lncRNA*Δ (Figure 1D). This suggests that the *lncRNA*Δ mutation abolishes expression of both lncRNAs, consistent with our prediction.

Next, we conducted transcriptional induction analysis as above using isogenic *dbp2*Δ and *dbp2*Δ* lncRNA*Δ cells to determine if the rapid induction phenotype is linked to the presence of the *GAL* lncRNAs. Strikingly, incorporation of the *lncRNA*Δ mutation in the *DBP2*-deficient strain restored the induction kinetics of both *GAL7* and *GAL10* to near wild-type profiles (Figure 1E–F). This suggests that the rapid induction of *GAL7* and *GAL10* from repressive conditions in *dbp2*Δ cells is lncRNA-dependent, indicating that the *GAL* lncRNAs play an as-of-yet uncharacterized role in gene activation. Alternatively, the delayed activation in *dbp2*Δ* lncRNA*Δ cells may be due to a role for Reb1 and/or the Reb1-binding sites in efficient expression of *GAL7* and *GAL10*.

### Defects in RNA Decay and Decapping Cause Rapid Induction of the *GAL* Cluster Genes from Repressive Conditions

Previous studies have utilized mutant strains with impaired RNA decay pathways to demonstrate the roles of lncRNAs at targeted gene loci [25], [38]. The 5′-3′ exonuclease, Xrn1, is required for degradation of both the *GAL10* and *GAL10s* lncRNAs [26], [38], [42], [43]. *DCP2*-deficient cells also accumulate lncRNAs but through a defect in RNA decapping [25]. Interestingly, up-regulation of the *GAL* lncRNAs, via loss of *DCP2*, has been linked to delayed transcriptional activation of the *GAL* genes from derepressed conditions (+raffinose) [25]. This was also observed for *xrn1*Δ cells, but to a lesser extent [25]. Recent studies have shown that both Dcp2 and Xrn1 are present in the nucleus and associate with transcribed chromatin, indicative of a direct link between decay and gene expression [44], [45]. However, contribution of RNA decay pathways to induction from repressed conditions (+glucose) has not been addressed.

**Figure 2:**
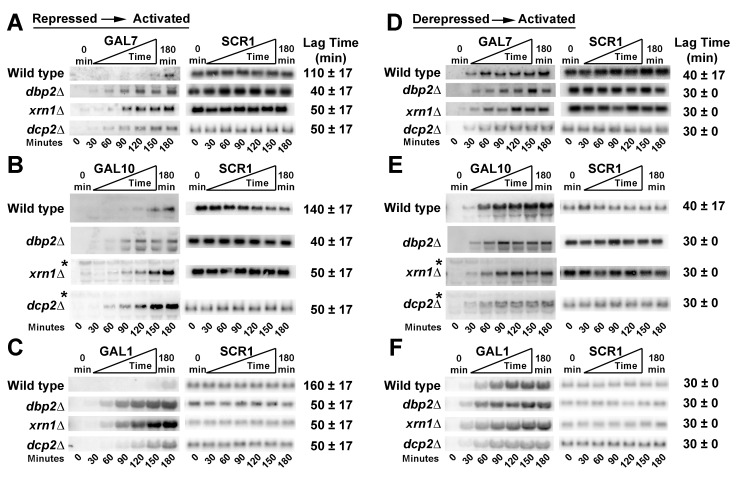
All three *GAL* cluster genes are rapidly induced from repressed conditions upon loss of *DBP2* or the RNA decay factors *XRN1* and *DCP2*. (A–C) *GAL7* (A), *GAL10* (B), and *GAL1* (C) are rapidly induced from repressed conditions in *dbp2*Δ, *xrn1*Δ, and *dcp2*Δ strains. Induction assays were conducted as in Figure 1 with isogenic *xrn1*Δ, *dbp2*Δ, *dcp2*Δ, and wild-type strains. Asterisks mark the *GAL10* lncRNA transcripts, which are visible in the *xrn1*Δ and *dcp2*Δ strains due to high abundance and the use of dsDNA probes (most visible from 0–90 min). Lag times represent the average of three biological replicates and the s.d. as in Figure 1. (D–F) Induction of *GAL7* (D), *GAL10* (E), and *GAL1* (F) from derepressed (+raffinose) conditions in *dbp2*Δ*, xrn1Δ, and dcp2Δ cells occurs with wild-type kinetics. Transcriptional induction was measured as above following a nutritional shift from derepressed (+raffinose) to activated (+galactose) conditions.*

To determine if the up-regulation of lncRNAs, via loss of RNA decay and/or decapping pathways, impacts the expression of the *GAL* genes from repressed conditions, we analyzed the transcriptional induction of *GAL7* and *GAL10* in *xrn1*Δ and *dcp2*Δ strains (Figure 2A–B). We also included *dbp2*Δ cells in this analysis for comparison to studies above. Surprisingly, and in contrast to defective expression, this revealed that *GAL7* and *GAL10* are rapidly induced in both *xrn1*Δ and *dcp2*Δ strains with overabundant lncRNAs. In fact, detectible transcripts appear 2- to 3-fold faster in these strains than in wild type, similar to the rapid induction kinetics of *dbp2*Δ cells (Figure 2A–B). Note that the *GAL10* lncRNA is also readily detectable in these RNA decay-deficient strains due to the use of a double-stranded DNA probe and consistent with the role of Xrn1 and Dcp2 in lncRNA decay (Figure 2B, asterisks) [25], [26], [38]. Thus, loss of genes encoding either the RNA helicase *DBP2* or the RNA decay factors *XRN1* or *DCP2* results in faster activation of the protein-coding *GAL* genes from repressive conditions. This suggests that the *GAL* lncRNAs may actually promote gene expression.

### *GAL1* Is Also Rapidly Activated from Repressed Conditions

In contrast to our results above, prior studies have proposed a repressive role for the *GAL10* lncRNA [24]–[26]. However, a major difference between our studies and past reports is that prior experiments were primarily focused on *GAL1* induction from derepressive conditions (+raffinose), rather than *GAL10* and *GAL7* from a repressive state (+glucose) [24]–[26]. To determine if *GAL1* exhibits a different induction profile than *GAL7* and *GAL10*, we analyzed the induction of this gene as above (Figure 2C). Northern blotting analysis of RNAs from wild-type, *dbp2*Δ, *xrn1*Δ, and *dcp2*Δ strains revealed that *GAL1* is also rapidly induced from repressive conditions in all three mutant strains with lag times of ∼50 min (Figure 2C). This suggests a common mechanism for the *GAL* lncRNAs at all three *GAL* cluster genes.

### Induction of the *GAL* Cluster Genes from Derepressive Conditions Occurs with Wild-Type Kinetics for *dbp2*Δ, *xrn1*Δ, and *dcp2*Δ Strains

In the presence of glucose, the *GAL* genes are repressed through several mechanisms, including the action of glucose-dependent transcriptional repressors (Figure 1A) [28], [31], [46]–[48]. However, when cells use raffinose as a carbon source, the *GAL* genes become derepressed due to environmentally induced loss of repressors (Figure 1A). To determine if the rapid induction of the *GAL* genes is specific for activation from repressive conditions (+glucose), we conducted induction analysis from the derepressed state (+raffinose). Interestingly, wild-type, *dbp2*Δ, *xrn1*Δ, and *dcp2*Δ strains all exhibited similar induction kinetics from derepressed to activated conditions with the appearance of transcripts within ∼30 min for all three *GAL* cluster genes (Figure 2D–F). This is consistent with a recent study showing that *xrn1*Δ cells accumulate *GAL7* and *GAL10* transcripts at the same rate as wild-type cells when induced from raffinose [45]. *DCP2*-deficient cells also displayed detectible transcripts at 30 min postinduction for all three *GAL* genes, albeit with an apparent reduction of transcript levels for *GAL1* as compared to wild type (Figure 2D–F, bottom). This demonstrates that the rapid induction of *GAL7*, *GAL10*, and *GAL1* is specific for the environmental switch from repressive (+glucose) to activating (+galactose) conditions. Moreover, it suggests that the loss of the RNA decay machinery does not necessarily result in lncRNA-dependent repression [25], [36].

### RNA Decapping Deficiencies Impair *GAL1* Transcript Accumulation

**Figure 3:**
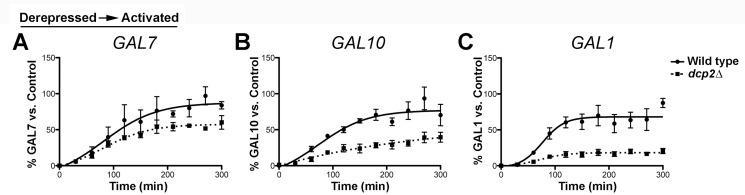
Loss of *DCP2* impairs *GAL1* transcript accumulation when induced from derepressive conditions. (A–C) Extended time course for analysis of *GAL7* (A), *GAL10* (B), and *GAL1* (C) induction from derepressed conditions in *dcp2*Δ cells. Wild-type and *dcp2*Δ cells were grown in raffinose as above and were shifted to galactose to induce transcription of the *GAL* cluster genes. RNA fractions were isolated at 30 min intervals over a 300 min time frame. Resulting transcript profiles from three biological replicates were normalized to *scR1* and plotted over time as a percentage of the average transcript levels with respect to a fully induced, wild-type “control” RNA for normalization between replicates. The “control” corresponds to total RNA isolated from wild-type cells after 5 h in galactose media following initial growth in raffinose for maximal expression. Error bars indicate the SEM. Statistical significance was calculated using a two-tailed *t* test. Time points with significantly different transcript levels (*p*<0.05) between wild-type and dcp2Δ cells for each gene are as follows: *GAL10*, 60–120 min time points; *GAL1*, 90–150, 240, 300 min time points. The 210 and 270 min time points for *GAL7* correspond to *p*<0.10, whereas no other time points in the *GAL7* analysis displayed significantly different transcript levels between wild-type and *dcp2*Δ cells.

Prior studies suggested that *GAL* lncRNAs are repressive based on defective induction of the *GAL* genes in RNA decapping and decay-deficient strains [25]. However, our results suggest that this is not the case for *xrn1*Δ cells with defective RNA decay. To determine if the apparent reduction in mRNA levels in *dcp2*Δ cells above indicates a specific requirement for decapping in *GAL* gene induction, we conducted longer induction analyses from derepressive conditions for three, independent biological replicates. We then graphed the resulting transcript levels over time as the fraction of a fully induced wild-type RNA sample (“Control”) following normalization to the *SCR1* loading control (Figure 3A–C). Consistent with previous studies, *dcp2*Δ cells displayed severe *GAL1* expression defects, with levels reaching only 20% of wild type after 5 h of induction (Figure 3C) [25]. *GAL10*, on the other hand, showed more moderate defects more in line with defective transcript accumulation than impaired initiation, whereas the *GAL7* induction profile was similar between wild-type and *dcp2*Δ cells (Figure 3A–B). This suggests that the decapping requirement for robust expression of lncRNA-targeted, inducible genes may be specific to *GAL1* [25], [36]. Furthermore, the fact that *dcp2*Δ cells show enhanced induction from repressed conditions (Figure 2A–C) argues against a generally repressive role for the *GAL* lncRNAs. Thus, the previously described lncRNA-dependent repression at the *GAL* cluster in RNA decay-deficient strains may reflect a requirement for decapping in the accumulation of *GAL1* transcripts, and especially *GAL1*, rather than a repressive role for the *GAL* lncRNAs.

### *DBP2-* and *XRN1*-Deficient Cells Display Faster Recruitment of RNA Polymerase II to *GAL7* and *GAL10* Genes

**Figure 4:**
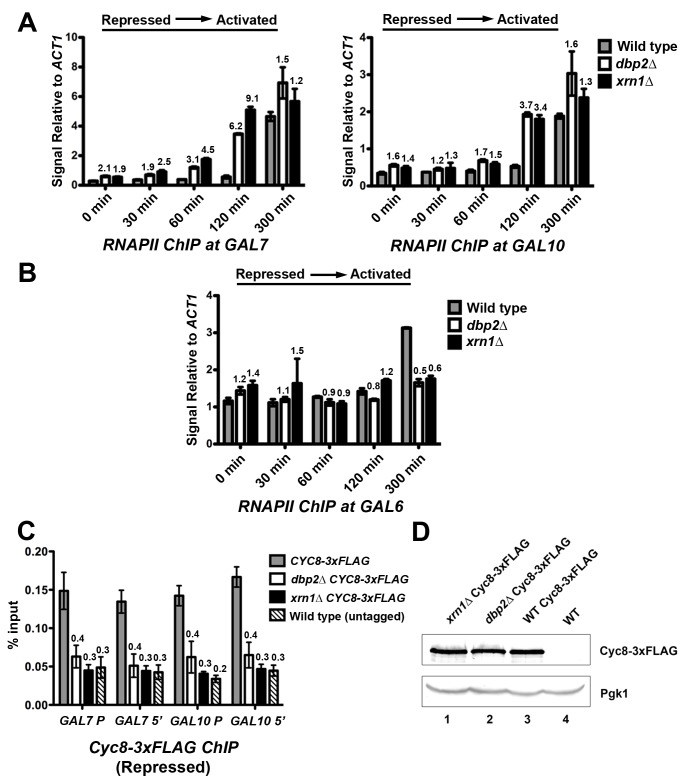
Rapid induction of the *GAL* genes correlates with faster recruitment of RNAPII and reduced corepressor binding to chromatin. (A) RNAPII is recruited faster to *GAL7* (left) and *GAL10* (right) promoters following a shift from repressive to activating conditions in *XRN1*- and *DBP2*-deficient cells. Wild-type, *dbp2*Δ, and *xrn1*Δ cells were shifted from transcriptionally repressive conditions (+glucose) to transcriptionally active conditions (+galactose). Cells were collected before (0 min) and at 30 min, 60 min, 120 min, and 300 min time points following a shift to galactose media. ChIP was conducted using an anti-Rpb3 antibody followed by qPCR. Results are presented as the relative Rpb3 occupancy at the *GAL10* or *GAL7* promoter with respect to the constitutively activated *ACT1* gene. Numbers above each bar represent the fold above wild type at the same time point postinduction for both *dbp2*Δ and *xrn1*Δ cells. (B) The galactose-dependent *GAL6* gene does not show increased RNAPII recruitment in *dbp2*Δ or *xrn1*Δ cells. ChIP was conducted as above followed by qPCR at *GAL6* promoter. Results are represented as the relative Rpb3 occupancy at the *GAL6* promoter with respect to the *ACT1* gene. (C) Both *dbp2*Δ and *xrn1*Δ cells display reduced association of the Cyc8 component of the Tup1–Cyc8 co-repressor complex at *GAL* genes under repressive conditions. Briefly, wild-type, *dbp2*Δ, and *xrn1*Δ cells harboring a *3×FLAG*-tagged *CYC8* at the endogenous locus as well as a wild-type strain with untagged *CYC8* were grown under transcriptionally repressive conditions (+glucose), representing the 0 min time point for the induction time courses above, and were then subjected to ChIP with anti-FLAG antibodies. Cyc8–3×FLAG occupancy is presented as the percentage of isolated DNA over input. Numbers above each bar represent the fraction of bound DNA in each strain versus that in the wild-type strain harboring the *3×FLAG-tagged CYC8*. (D) Cyc8–3×FLAG is expressed at similar levels in wild-type, *dbp2*Δ, and *xrn1*Δ strains. Western blotting was conducted with whole cell lysates from the indicated strains and Cyc8–3×Flag was detected with polyclonal anti-FLAG antibodies. Pgk1 serves as a loading control, whereas wild type with an untagged Cyc8 (lane 4) demonstrates antibody specificity.

Our results above provide evidence that the *GAL* lncRNAs may act in a positive manner by stimulating induction of the protein-coding *GAL* genes from repressed conditions. However, it is also possible that the increase in transcript abundance over time is due to a decrease in mRNA decay rather than an increase in transcriptional activity. To determine if the rapid induction correlates with an increased rate of transcriptional induction in *dbp2*Δ and *xrn1*Δ cells as compared to wild type, we asked if RNA polymerase II (RNAPII) is recruited faster to the *GAL7* and *GAL10* gene promoters [39], [49]. RNAPII recruitment was measured by conducting chromatin immunoprecipitation (ChIP) over a 300-min time course following induction from repressed conditions with an antibody to a RNAPII core subunit (anti-Rpb3) (Figure 4A). Suggestive of a transcriptional effect, this revealed that RNAPII is recruited to the *GAL7* and *GAL10* promoters more rapidly in both *dbp2*Δ and *xrn1*Δ cells (Figure 4A). This faster recruitment was most evident at 120 min postinduction, with ∼4-fold and ∼6- to 9-fold higher levels of RNAPII at *GAL7* and *GAL10*, respectively (Figure 4A). This suggests that loss of *DBP2* or *XRN1*, and the resulting accumulation of the *GAL* lncRNAs, results in a direct effect on transcription initiation. In contrast, analysis of the galactose-inducible *GAL6* gene revealed similar RNAPII recruitment rates for all three strains with a slightly lower RNAPII signal for *xrn1*Δ and *dbp2*Δ cells at the 300 min time point (Figure 4B) [50]. The latter is consistent with recent studies showing that *xrn1*Δ cells have reduced steady-state transcription levels [45]. Furthermore, it demonstrates that the rapid recruitment of RNAPII is specific for the *GAL* lncRNA-targeted genes within the *GAL* cluster.

### Derepression of *GAL7* and *GAL10* Correlates with Reduced Binding of the Cyc8 Corepressor

Glucose-dependent repression is accomplished by transcription factors Mig1, Mig2, and Nrg1, which recognize specific DNA sequences and subsequently recruit co-repressor complexes like the Tup1–Cyc8 complex [28], [40], [46]–[48], [51], [52]. To determine why *dbp2*Δ and *xrn1*Δ cells exhibit faster recruitment of RNAPII, we asked if these strains display lower levels of bound co-repressors. To test this, we conducted ChIP assays to measure the association of Cyc8 at *GAL7* and *GAL10* at the 0 min time point when the *GAL* genes are repressed. We tested both the promoter and 5′ end of *GAL7* and *GAL10* as Tup1 has been shown to associate with the ORF and the promoter of specific gene loci [53]. Consistent with the more rapid recruitment of RNAPII, both *DBP2-* and *XRN1*-deficient cells exhibited severely reduced Cyc8 binding at both the promoter and 5′-end of the open reading frame (ORF), with levels equivalent to background ChIP signal (Figure 4C). Western blotting revealed that *CYC8–3×FLAG* is expressed at similar levels in all three strains, indicating that reduced binding is not due to different protein levels (Figure 4D). Thus, the rapid induction of *GAL7* and *GAL10* in *xrn1*Δ and *dbp2*Δ cells correlates with reduced association of Cyc8 corepressor. This provides an explanation for the rapid induction of *GAL* gene expression from the repressed (+glucose) but not derepressed (+raffinose) conditions (Figures 1 and 2); the *GAL* genes are derepressed in the *dbp2*Δ and *xrn1*Δ strains.

### The *GAL* lncRNAs Do Not Alter the Transcriptional Induction Profiles of *GAL7* or *GAL10* from Derepressed Conditions in *XRN1-*Deficient Cells

**Figure 5:**
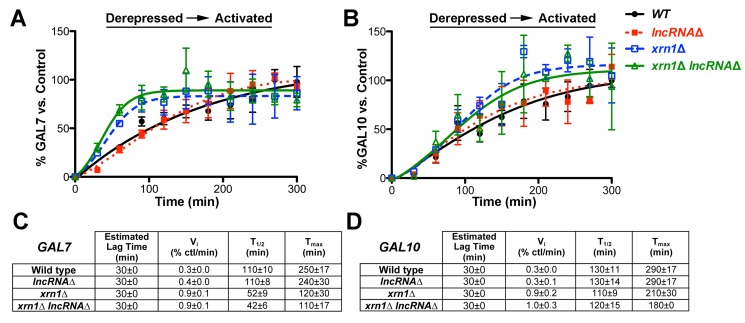
The *GAL* lncRNAs do not alter the *GAL7* or *GAL10* transcription profile in *xrn1*Δ cells when induced from derepressed conditions. (A–B) The *xrn1*Δ and *xrn1*Δ* lncRNA*Δ strains display superimposable transcriptional induction profiles of *GAL7* (A) and *GAL10* (B) from derepressed conditions. Isogenic wild-type (closed black circle), *lncRNA*Δ (closed red square), *xrn1*Δ (open blue square), and *xrn1*Δ* lncRNA*Δ (open green triangle) strains were analyzed for both rapid induction from derepressive conditions (+raffinose) and final, steady-state transcript levels by conducting time courses as above up to 300 min. Resulting induction profiles were plotted as in Figure 3 following normalization to a fully induced *GAL* “control” and to *SCR1*. Representative northern blots are shown in Figure S1. (C–D) *GAL7* (C) and *GAL10* (D) transcriptional induction kinetic profiles are similar between *xrn1*Δ and *xrn1*Δ *lncRNA*Δ cells. The lag times were calculated as above for each individual biological replicate following normalization to *SCR1* and are reported as the average with s.d. The T_max_ and T_1/2_ correspond to the time point when transcript levels plateau and the half-time to T_max_, respectively. Initial velocities were calculated as the slope of the straight line from the lag time to T_max_, with increases most likely reflecting greater transcript production in a given cell population over time. All kinetic parameters were calculated independently for each biological replicate after graphical analysis, after normalization to *SCR1* and the control RNA, and are the average of the three replicates with the s.d.

If derepression is caused by the *GAL* lncRNAs, then deletion of these noncoding RNA molecules should have no effect on the induction or final levels of *GAL7* and *GAL10*. To determine if this is the case, we constructed *xrn1*Δ and *xrn1*Δ* lncRNA*Δ cells, as *xrn1*Δ and *dbp2*Δ cells exhibit similar induction profiles. We then conducted extended time courses of wild-type, *xrn1*Δ, *lncRNA*Δ, and *xrn1*Δ* lncRNA*Δ strains to measure both the induction kinetics and steady-state transcript levels of the *GAL* genes from the derepressed (+raffinose) condition (Figure S1, representative northern blot). Resulting induction profiles were then graphed for each condition, lag times were determined as above, and the velocity of transcript accumulation and final steady-state levels were determined after normalization to *SCR1* and with respect to a fully induced, wild-type strain (“control”) (Figure 5).

Consistent with our shorter time course analysis (Figure 2), both wild-type and *xrn1*Δ cells displayed similar lag times for induction and final steady-state transcript levels for both *GAL7* and *GAL10* when induced from derepressive conditions (+raffinose) (Figure 5A–B). This is in line with other studies demonstrating identical induction profiles from derepressive conditions between wild-type and *xrn1*Δ cells [26], [45]. Moreover, this further illustrates that *GAL* lncRNA-dependent repression is not a general phenotype of RNA decay-deficient strains. *XRN1*-deficient cells did, however, show increased transcript levels at early time points within the induction profile of both genes, as evidenced by the higher “shoulder” in the graphical analysis (Figure 5A–B) and increased initial velocities of transcript accumulation (Figure 5C–D). These increases are not due to the *GAL* lncRNAs though, as the induction profiles of *GAL7* and *GAL10* in the *xrn1*Δ strain are superimposable with the *xrn1*Δ* lncRNA*Δ strain. This also demonstrates that the *lncRNA*Δ mutation itself, and resulting loss of Reb1 binding, does not impair the transcriptional activity of *GAL7* or *GAL10*. Consistently, both the *xrn1*Δ and *xrn1*Δ* lncRNA*Δ strains have similar kinetic parameters for transcriptional induction. This includes identical initial velocities as well as half time (T_1/2_) and time to maximum transcript levels (T_max_) between *xrn1*Δ strains regardless of the presence or absence of the *GAL* lncRNAs (Figure 5C–D). Thus, the *GAL* lncRNAs do not alter the transcriptional induction of the *GAL* genes in *XRN1*-deficient cells from derepressive conditions.

### The *GAL* lncRNAs Alter the Kinetics of Induction from Repressed Conditions in *xrn1*Δ Cells

**Figure 6:**
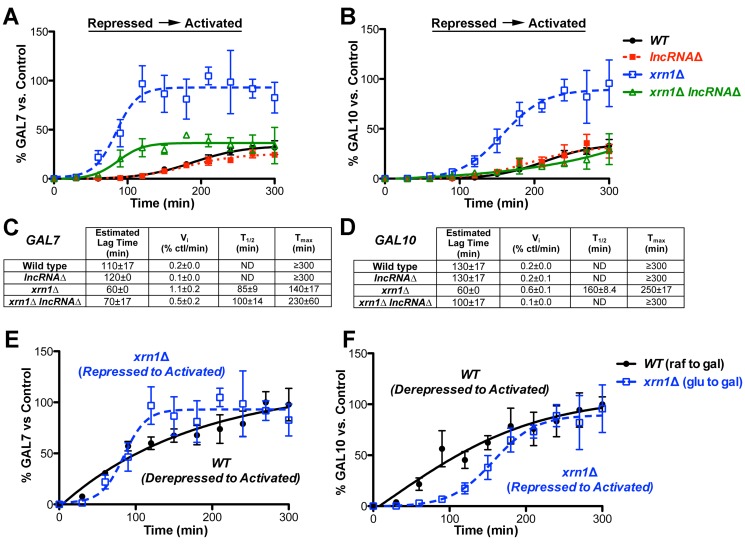
The *GAL* lncRNAs enhance the kinetics of transcriptional induction from repressed conditions in *xrn1*Δ cells. (A–B) Rapid induction of *GAL7* (A) and *GAL10* (B) transcripts in *xrn1*Δ cells is lncRNA-dependent. Transcriptional induction of isogenic wild-type (closed black circle), *lncRNAΔ* (closed red square), *xrn1*Δ (open blue square), and *xrn1*Δ* lncRNA*Δ (open green triangle) strains induced from repressed conditions (+glucose) was analyzed as above to determine lag times, initial velocities, and final levels. Note that the transcript abundance is reported as a percentage of the fully induced “control” as in Figure 5, illustrating that wild-type cells are not fully induced at the end of this time course. Representative northern blots are shown in Figure S2. (C–D) *GAL7* (C) and *GAL10* (D) transcriptional induction kinetic profiles illustrate lncRNA-dependent kinetic enhancement from repressed conditions. Kinetic parameters were determined as in Figure 5. Strains that did not reach an induction plateau within the 300 min time frame display T_max_ values that are equal to or greater than 300 min. In these cases, half-times (T_1/2_) were not determined (ND). (E–F) The lncRNA-dependent enhanced induction in *xrn1*Δ cells parallels wild-type induction from a derepressed state. Transcriptional profile overlay of *GAL7* (E) and *GAL10* (F) induction in wild-type cells (closed black circle) from derepressed to activated conditions as compared to *xrn1*Δ cells (opened blue square) from repressive conditions.

We then analyzed the transcriptional induction kinetics of *xrn1*Δ cells from repressed (+glucose) to activated conditions to determine the role of the *GAL* lncRNAs during this specific transcriptional switch (Figure S2, representative northern blot). Resulting *GAL7* and *GAL10* profiles were plotted as above with respect to the same, fully induced wild-type control. In contrast to induction from derepressed conditions, this analysis revealed sharply different transcriptional profiles between *xrn1*Δ and wild-type cells (Figure 6). In fact, *xrn1*Δ cells showed shorter lag times as well as ∼3-fold higher levels of *GAL7* and *GAL10* transcripts as compared to wild type (Figure 6A–B). Kinetic analysis revealed that *xrn1*Δ cells have a more rapid approach to steady state than wild-type cells when induced from repressive conditions, as evidenced by the reduced lag time and 3- to 6-fold increase in the initial rate (V_i_) of transcript accumulation for both *GAL7* and *GAL10* (Figure 6C–D). This is also illustrated by the fact that *xrn1*Δ cells reach 100% of the fully induced “control” within the 300 min time frame, while wild-type cells do not (Figure 6A–B). This rapid, high-level induction in *xrn1*Δ cells during the switch from repressed to activated conditions is consistent with the reduced association of Cyc8 and faster recruitment of RNAPII (see Figure 4).

Strikingly, removal of the *GAL* lncRNAs abolished both the rapid induction and high transcript levels in the *xrn1*Δ strain, resulting in profiles more similar to wild type (Figure 6A–B). In fact, the *GAL10* induction profile of *xrn1*Δ* lncRNA*Δ cells is superimposable with that of wild-type cells, demonstrating that the rapid induction of this gene in *xrn1*Δ cells is fully dependent on the *GAL* lncRNAs (Figure 6B,D). The induction profile of *GAL7* was also restored by incorporation of the *lncRNA*Δ mutation into the *xrn1*Δ strain, but to a lesser extent (Figure 6A,C). This partial reduction may be due to the contribution of another lncRNA that overlaps *GAL7*, as prior studies have indicated the presence of a *GAL7* antisense transcript that originates outside of the *lncRNA*Δ mutation [24]. Interestingly, removal of the *GAL* lncRNAs resulted in both a longer lag time as well as decreased initial velocity in *XRN1*-deficient cells (Figure 6C–D). This suggests that the *GAL* lncRNAs function at the kinetic level by enhancing the approach to steady state. It also indicates that the *GAL* lncRNA molecules have the largest impact at the point of induction of the protein-coding *GAL* genes.

Next, we asked if the induction of *xrn1*Δ cells from repressed conditions (+glucose) is similar to that of wild-type cells from derepressed conditions (+raffinose), with the idea that lncRNA-dependent derepression in *XRN1*-deficient cells should mimic the derepressed transcriptional state in wild-type cells. Overlaying the *GAL7* and *GAL10* induction profiles revealed that *xrn1*Δ cells exhibit a similar induction trend from repressed conditions as wild-type cells induced from derepressed conditions (Figure 6E–F). This is consistent with the fact that *xrn1*Δ cells have reduced association of Cyc8 (Figure 4) and the idea that the *GAL* lncRNAs promote derepression of the protein-coding *GAL* genes in *xrn1*Δ cells. The difference in shape of the two curves between wild type and *xrn1*Δ may reflect the activity of other, glucose-dependent repression mechanisms (see Discussion) or the presence of low levels of Cyc8 at the *GAL* gene promoters that are below our detection by ChIP. Regardless, this is consistent with a model whereby the *GAL* lncRNAs activate gene expression by promoting derepression.

### The *GAL* LncRNAs Promote Induction of *GAL7* and *GAL10* Genes from Repressed Conditions in Wild-Type Cells

**Figure 7:**
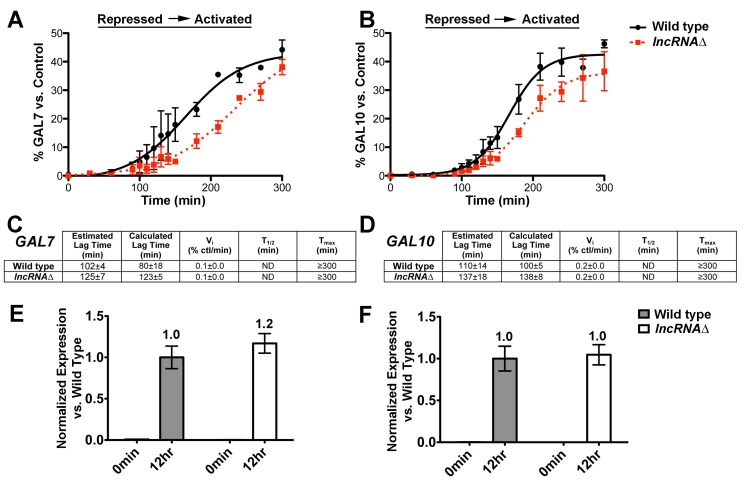
The *GAL* lncRNAs kinetically enhance *GAL* gene induction from repressed conditions in wild-type cells. (A–B) The *GAL* lncRNAs increase the rate of *GAL7* and *GAL10* activation in wild-type cells. Graphical representation of transcriptional induction of *GAL7* (A) and *GAL10* (B) in wild-type (closed black circle) and *lncRNAΔ* (closed red square) strains from repressed to activated conditions. High-resolution transcriptional analysis was conducted with wild-type or *lncRNA*Δ cells from repressed conditions from 0 to 300 min by including 10 additional 10-min time points between 90 and 150 min. Transcript abundance is reported as a percentage of the control as previously described. The differences in final *GAL7* or *GAL10* transcript levels at the 300 min time point are not statistically significant (*p* value>0.2). Representative northern blots are shown in Figure S3. (C–D) The GAL lncRNAs increase the kinetics of transcriptional activation from repressive conditions. Transcription induction parameters for the wild-type and *lncRNA*Δ strains were determined as above for three independent biological replicates. Calculated lag times were determined using curve-fitting analysis (DM Fit v. 2.0) [54], which facilitates quantitative assessment of lag from the curve fit (Figure S4). Lag times assessed from the data points as in prior figures are denoted as “estimated” lag times for differentiation from the curve fitting values. The estimated lag times result in *p* values from a two-tailed *t* test of 0.12 and 0.09 for *GAL7* and *GAL10*, respectively, whereas calculated lag times are significantly different between wild-type and *lncRNA*Δ strains (*GAL7* lag *p* value = 0.01; *GAL10* lag *p* value = 0.07). The initial velocities of transcript accumulation are not significantly different. (E–F) The presence of *GAL* lncRNAs does not alter the final levels of *GAL7* and *GAL10* transcripts at longer time points postactivation. *GAL7* (E) and *GAL10* (F) transcript levels were measured by RT-qPCR under repressed conditions (0 min time point) and after a 12-h shift to activated conditions (12-h time point) from repressed to activating conditions. *GAL7* and *GAL10* transcripts were measured from three biological replicates and normalized to *ACT1*. Normalized expression is presented as the average fold change from the first wild-type biological replicate with error bars representing the SEM. Note that the *GAL10* lncRNA, which is also recognized by the *GAL10* primers, is not evident at the 0 min time point due to the high expression levels of *GAL7* and *GAL10* after 12 h and necessary scaling of the bar graph.

Our results above demonstrate a positive role for the *GAL* lncRNAs in promoting gene expression. Furthermore, our studies suggest that these noncoding RNAs impact the timing of transcriptional activation by stimulating the kinetics of induction. Given this knowledge, we then asked if the *GAL* lncRNAs have any effect in wild-type cells, which were not initially evident due to the analysis of induction with 30 min time points. To this end, we conducted a higher time-resolved analysis of *GAL7* and *GAL10* induction from repressed conditions in wild-type and *lncRNA*Δ cells by including additional 10 min time points at the induction point, immediately prior to and following recruitment of RNAPII (Figure 4A). Strikingly, this revealed distinct *GAL7* and *GAL10* induction profiles between wild-type and *lncRNA*Δ strains (Figure 7A–B; Figure S3). More specifically, wild-type cells expressing the *GAL* lncRNAs induced both *GAL7* and *GAL10* faster than the *lncRNA*Δ cells, resulting in a clear separation of the transcriptional profiles between the two strains along the *x*-axis (Figure 7A–B). Lag time calculation revealed that *lncRNA*Δ cells lacking the *GAL* lncRNAs exhibit transcriptional lags of ∼125–137 min, and wild-type cells induced both *GAL7* and *GAL10* ∼30 min faster (Figure 7C–D, estimated lag time). This suggests that the *GAL* lncRNAs promote induction in wild-type cells. To more quantitatively assess lag times between wild-type and *lncRNA*Δ strains, we then utilized a curve fitting method for mathematical assignment of lag times (DM fit v2.0 Excel Macro) [54], which was only possible with higher time-resolved analysis (Figure S4). The calculated lag times, although similar in magnitude to the estimates, resulted in more statistically significant differences between wild-type and *lncRNAΔ* strains (Figure 7C–D; *p* values<0.1 for both genes). This suggests that curve fitting may be a more accurate assessment of lag times from biological data sets. More importantly, however, this demonstrates that the *GAL* lncRNAs promote a subtle but reproducible acceleration of induction in wild-type cells. In contrast to the lncRNA-dependent reduction of lag times, we did not observe a significant difference in the initial velocity of transcript accumulation between strains, however (Figure 7C–D). This indicates that either the *GAL* lncRNAs do not alter transcript accumulation rates in wild-type cells or that this effect is not evident by analysis across a cell population when the lncRNA levels are low (est. 1 in 14 cells in [24]). Regardless, the statistically significant shift in lag times suggests that the *GAL* lncRNAs enhance the induction of the *GAL7* and *GAL10* genes in wild-type cells, consistent with an effect on induction kinetics rather than steady-state levels. Moreover, the final levels of *GAL7* and *GAL10* within the 5-h time course (Figure 7A–B) or after 12 h postinduction were not significantly different between wild-type and *lncRNA*Δ strains (Figure 7E–F). This indicates that the *GAL* lncRNAs promote transcriptional induction in wild-type cells without altering the final transcript abundance of the targeted protein-coding genes. We propose that the *GAL* lncRNAs poise the protein-coding *GAL* genes for rapid induction, thereby enhancing the transcriptional switch from repressed to activated conditions.

**Figure 8:**
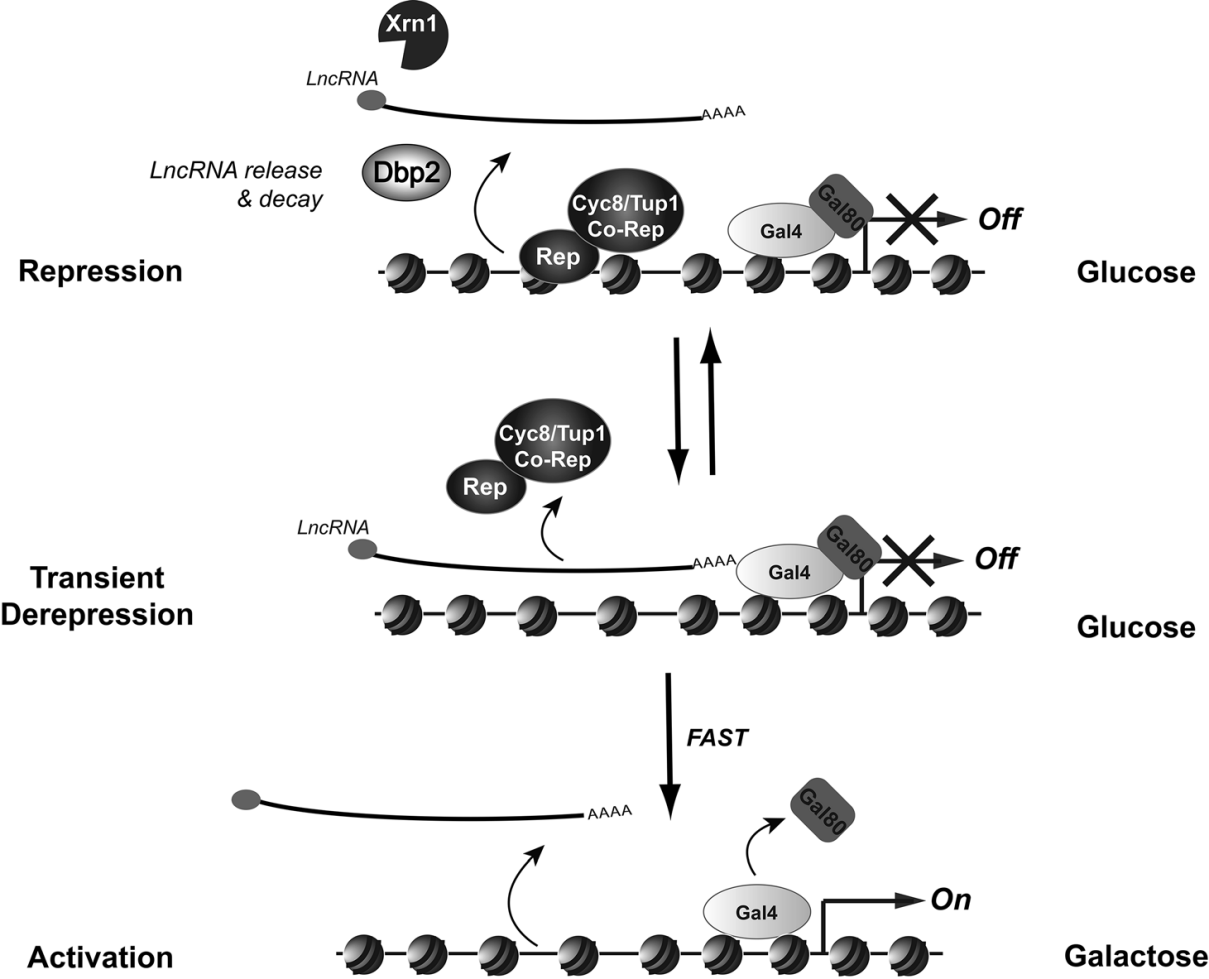
The *GAL* cluster lncRNAs poise the protein-coding *GAL* genes for rapid induction from repressive conditions. Transcriptional repression of the protein-coding *GAL* genes occurs through binding of glucose-responsive transcriptional repressors (Rep) and subsequent recruitment of co-repressors Tup1–Cyc8 to gene promoters (repression) [28], [32], [46]–[48], [51]. Derepression is accomplished through lncRNA-dependent displacement of these repressors from chromatin. Displacement may occur through transcriptional interference and/or formation of RNA–DNA hybrids between the lncRNA and targeted, protein-coding gene. Derepression is transient, however, due to the action of Dbp2 and Xrn1, which facilitate lncRNA release from chromatin and RNA decay, respectively. This equilibrium between repressed and derepressed states allows for faster transcriptional activation in the presence of galactose. Activation then requires release of the Gal80 inhibitor protein from the Gal4 activator and subsequent recruitment of coactivating complexes and RNAPII (not pictured) [29]. Thus, the *GAL* lncRNAs function at the temporal level of gene regulation by enhancing the kinetics of *GAL* gene induction from transcriptionally repressive conditions.

Taken together, our studies demonstrate that the *GAL* lncRNAs enhance the activation kinetics of the inducible *GAL* genes from repressed conditions. Based on these observations, we present a model whereby the *GAL* lncRNAs displace glucose-dependent repressors from the *GAL* gene promoters under typically repressive conditions (Figure 8). Because this role does not result in full derepression in wild-type cells, we suggest that this displacement is transient due to the action of Dbp2 and Xrn1, which promote lncRNA release and decay, respectively [37], [38], [43], [55]. If this is the case, this suggests that the *GAL* lncRNAs complement the roles of proteinaceous factors to increase the efficiency of the *GAL* gene transcriptional switch [29], [39], [56]. Moreover, these studies indicate that the *GAL* lncRNAs promote formation of a dynamic chromatin template. These dynamics facilitate faster activation by poising the *GAL* genes for induction in response to galactose, which may provide a selective advantage for cells responding to changing environmental conditions. This indicates that the *GAL* lncRNAs temporally regulate gene expression by influencing the rate of transcriptional responses to extracellular stimuli.

## Discussion

In an effort to define the role of the *GAL10s* lncRNA at the *GAL* cluster, our studies uncovered an important new role for both this lncRNA and the previously characterized *GAL10* lncRNA in activating gene expression from repressed conditions [24]–[26]. Glucose-dependent repression of the *GAL* genes is accomplished through several, mechanistically distinct processes including inhibition of the Gal4 activator, reduction of intracellular galactose uptake, and transcriptional repression of *GAL* promoters [28], [31], [34], [40], [46], [48], [51], [56]. Our studies suggest that the *GAL* lncRNAs act on the latter mechanism by transiently displacing repressors from bound promoters, eliciting a dynamic equilibrium between derepressed and repressed states (Figure 8). We predict that this equilibrium poises the *GAL* genes for rapid induction, enhancing the transcriptional switch in response to extracellular signals.

The role of the *GAL* lncRNAs in enhancing induction is distinctly different from a true role in transcriptional activation, as has been documented for the roX RNAs in Drosophila or the activating ncRNAs (ncRNA-a) in mammalian cells [10], [57]. Instead, our studies are more consistent with an interference-based model, whereby the *GAL* lncRNAs prevent the association of transcription factors with targeted gene promoters. This is supported by our observation that the *GAL* lncRNAs promote derepression by reducing the association of Cyc8 with the *GAL* genes. It is also in line with the fact that the *GAL* genes are not activated by the *GAL* lncRNAs per se but that the rate of induction is faster. It is also important to note that the kinetics reported here reflect the average transcriptional profile across a cell population and not the profile of individual cells. Because the abundance of the *GAL* mRNAs varies widely across single cells during early induction [58], it is possible that the lncRNA-dependent derepression proposed here facilitates a more robust mRNA accumulation in individual cells. Alternatively, the *GAL* lncRNAs may allow a larger population of cells to rapidly “switch” from repression to activation. Recent studies of the antisense *PHO84* lncRNA have proposed such a model whereby synthesis of this lncRNA results in cellular heterogeneity within a culture, with a fraction of cells exhibiting lncRNA-dependent repression of sense *PHO84* expression [59].

One of the most surprising aspects of our findings is that the *GAL10* lncRNA was thought to be exclusively repressive [24], [25]. Although our studies show that both the *GAL10* and *GAL10s* lncRNAs promote gene expression, this is not necessarily mutually exclusive with a repressive role under specific conditions. However, it should be noted that the mechanism by which *GAL* lncRNAs induce transcriptional repression is still unknown. Early analysis of the *GAL10* lncRNA reported a delay of induction in a mixed glucose/galactose carbon source, making mechanistic insight difficult due to simultaneous presence of repressors and activators [24]. Subsequent studies then suggested that the *GAL* lncRNAs are repressive based on defective induction of the *GAL* genes in RNA decapping and decay-deficient strains [25]. While our studies corroborate the requirement for decapping for normal expression of the *GAL1*, and to a lesser extent *GAL10*, the fact that *xrn1*Δ cells do not show expression deficiencies and that both *xrn1*Δ and *dcp2*Δ cells show enhanced induction from repressed conditions argues against a repressive model. Instead, it is more likely that both the apparent expression defects in *dcp2*Δ cells and enhanced transcriptional induction occur through a common mechanism, whereby the *GAL* lncRNAs simply occlude transcription-factor binding sites at the targeted promoters. These transcription factors include glucose-dependent repressors when the *GAL* genes are induced from repressive conditions. However, the high level of the *GAL* lncRNAs in *dcp2*Δ cells may also cause interference with Gal4 or transcriptional coactivators such as SAGA and/or Mediator. This model would account for both the decreased transcriptional activity and histone acetylation at targeted chromatin (Figure 3) [25]. It is not clear, however, why *GAL1* is more sensitive to loss of decapping than other genes within the *GAL* cluster. Alternatively, the decreased transcriptional activity in *dcp2*Δ cells may be due to the recently proposed, and as-of-yet uncharacterized, role for decapping and decay factors in transcription [45]. Nevertheless, the fact that ablation of the *GAL10* lncRNA rescues *GAL1* transcriptional delays indicates that at least some part of the expression defect in *dcp2*Δ cells is dependent on the lncRNA [25]. Interestingly, the Set3C histone deactylase complex has also been shown to influence the kinetics of inducible genes [60], suggesting that lncRNA-dependent gene expression involves a complex interplay between histone modifications, lncRNAs, and metabolic genes.

One mechanism for promoter occlusion by lncRNAs is the formation of transient lncRNA–DNA hybrids at the *GAL* gene promoters. RNA–DNA hybrids, or R loops, are found in all organisms from yeast to humans and have been recently linked to regulation of chromatin rchitecture [61]–[63]. These structures form during transcription and have historically been associated with defects in termination and/or mRNP assembly (for review, see [63]). However, recent studies have found widespread formation of RNA–DNA hybrids at multiple gene loci in normal cells, with roles linked to transcriptional regulation, termination replication, and recombination [63]–[65]. Interestingly, the mammalian *DHFR* lncRNA forms an RNA–DNA triplex at the *DHFR* promoter [23]. This lncRNA represses transcription of the *DHFR* gene by interfering with the association of the TFIIB basal transcription factor, demonstrating that formation of this RNA–DNA hybrid occludes the binding site for the transcriptional apparatus. Although not an R loop, this study is consistent with the idea that lncRNAs may act through base pairing with target DNA. Recent studies implicating Dbp2 in both co-transcriptional mRNP assembly and in termination of coding and noncoding RNAs [37], [55], two processes that prevent RNA–DNA hybrid formation, is also suggestive of a role for these nucleic acid structures in *GAL* gene induction. This model may even account for transcriptional interference of *GAL7*, and reduced association of the Gal4 activator, in strains with defects in *GAL10* transcriptional termination [66], [67]. Moreover, recent work from the Tollervey lab has revealed striking differences in the termination/3′-end formation pathways and assembly of mRNA export factors between the majority of lncRNAs as compared to mRNAs, suggesting that the function of a transcript may be largely determined at late maturation steps [68]. The fact that p68, the human ortholog of Dbp2, also functions in lncRNA-dependent gene regulation suggests that the role for Dbp2 in RNA-mediated transcriptional control may be conserved between yeast and multicellular eukaryotes [69], [70].

Due to predominantly cytoplasmic localization [71]–[73], both Xrn1 and Dcp2 were long thought to function solely in cytoplasmic RNA decay. However, studies of noncoding RNAs implicated both of these factors in nuclear RNA decay, as loss of either gene product elicited transcriptional defects [25], [38], [74]. The Choder laboratory has now provided evidence that both of these RNA decay factors are present in the nucleus and associate with chromatin [45]. Although it was suggested that these RNA decay factors promote transcription through an as-of-yet uncharacterized mechanism, it is possible that Xrn1 and Dcp2 function in co-transcriptional RNA decay. If this is the case, RNA–DNA hybrids may accumulate in *xrn1*Δ and *dcp2*Δ strains as a result of failure to “clear” aberrant transcriptional products. This would be consistent with prior studies showing that the *GAL10* lncRNA functions in *cis* by suggesting that these decay enzymes also function at the site of synthesis [24].

Given that the *GAL* lncRNAs promote induction, one might ask why we do not observe a net increase in steady-state transcript levels. This is consistent with studies of the Set3C complex, whose loss results in altered *GAL* gene induction kinetics with no effect on the final transcript levels [60]. Moreover, this is a well-known phenomenon in pre–steady state enzyme kinetics, which depends on different mechanisms than steady state [75]. In the case of *GAL7* and *GAL10* expression, steady state is the period when the rate of RNA synthesis and decay are matched. Pre–steady state, however, is governed by release of transcriptional repressors and recruitment of RNAPII. Our data strongly suggest that it is these latter two processes that are likely accelerated by the *GAL* lncRNAs.

The idea that lncRNAs play a kinetic role was initially put forth by studies of the *PHO5* lncRNA, which promotes transcriptional activation of the *PHO5* gene by altering the rate of chromatin remodeling [15]. It is well established that the protein-coding genes within the *GAL* cluster are highly regulated through carbon-source-specific transcription factors [27], [29], [32], [40]. Upon a switch to galactose, glucose-dependent transcription factors are shunted to the cytoplasm, and the transcriptional activator Gal4 is released from the Gal80 inhibitor (Figure 7) [28], [32], [56], [76]. Our studies now show that the *GAL* lncRNAs add to this mechanism by promoting this transcriptional switch. This suggests that lncRNAs promote “kinetic synergism,” which is a model stating that kinetic alterations can have greater, combined effects on transcription than thermodynamics alone [77]. Kinetic synergism describes how the combination of multiple slow steps in transcriptional induction results in a more rapid and effective transcriptional activation. The *GAL* lncRNAs would function by promoting a more dynamic chromatin template, which synergistically enhances the activity of transcription factors by allowing transient access to DNA.

Our studies now complement the current knowledge regarding the function of lncRNAs by demonstrating that lncRNAs can influence the rate of transcriptional responses to extracellular cues. This is an exciting possibility because it suggests that the presence of lncRNAs may confer a selective advantage for a given organism to rapidly adapt to changing conditions. For example, wild-type cells could begin utilizing galactose as a carbon source at least 30 min earlier than cells without the *GAL* lncRNAs (Figure 7). This ability to influence the timing of a transcriptional switch would provide a rationale for the presence of lncRNAs in all eukaryotes and the conservation of these molecules near developmentally regulated genes in multicellular organisms [13], [17], [18], [21], [22]. Moreover, the ability of lncRNAs to alter chromatin dynamics may provide a more universal, functional role for widespread transcription of these noncoding molecules. Analysis of temporal effects of lncRNAs in multicellular organisms represents a future challenge in deciphering the role of these multifunctional regulators of the eukaryotic genome.

## Materials and Methods

### Plasmids and Strains

All plasmids were constructed by standard molecular biology techniques and are listed in Table 1. Yeast strains were constructed using classical yeast genetic techniques and are listed in Table 2. Oligos for PCR-mediated homologous recombination are listed in Table 3.

**Table 1:** Template plasmids for northern blot probes and strain construction.

Name	Description	Source/Reference
pGAL1-GAL10-GAL7	pYGPM11l14	Open Biosystems
pSCR1	pYGPM29b01	Open Biosystems
pUG6	KanMx disruption cassette plasmid	[78]
pAG32	HygB disruption cassette plasmid	[79]
p3×FLAG	p3×FLAG:KanMX	[80]

**Table 2:** Yeast strains.

Strain	Genotype	Source
Wild type (BY4741)	*MATa his3Δ1 leu2Δ0 met15Δ0 ura3Δ0*	Open Biosystems
*xrn1*Δ	*MATa xrn1::KanR his3Δ1 leu2Δ0 met15Δ0 ura3Δ0*	Open Biosystems
*dbp2*Δ* (BTY115)*	*MATa dbp2::KanR ura3Δ0 leu2Δ0 his3Δ0 met15Δ0 lys?*	[37]
*dcp2*Δ* (BTY289)*	*MATa dcp2::HygR his3Δ1 leu2Δ0 met15Δ0 ura3Δ0*	This study
Wild type (FT4)	*MATa ura3–52 trp1-Δ63 his3-Δ200 leu2::PET56*	[24]
*FT4+Reb1BS*Δ	*MATa ura3–52 trp1-Δ63 his3-Δ200 leu2::PET56 gal10::URA3::pMV12 (EcoRI/XhoI-Reb1 BSΔ with BS2 silent)*	[24]
*FT4 dbp2*Δ* (BTY219)*	*MATa ura3–52 trp1-Δ63 his3-Δ200 leu2::PET56 dbp2::KanR*	This study
*FT4+Reb1BS*Δ* dbp2*Δ* (BTY220)*	*MATa ura3–52 trp1-Δ63 his3-Δ200 leu2::PET56 gal10::URA3::pMV12 (EcoRI/XhoI-Reb1 BSΔ with BS2 silent) dbp2::KanR*	This study
*FT4 xrn1*Δ* (BTY226)*	*MATa, ura3–52, trp1-Δ63, his3-Δ200, leu2::PET56 xrn1::HygR*	This study
*FT4+Reb1BS*Δ* xrn1*Δ* (BTY227)*	*MATa, ura3-52, trp1-Δ63, his3-Δ200, leu2::PET56 gal10::URA3::pMV12 (EcoRI/XhoI-Reb1 BSΔ with BS2 silent) xrn1::HygR*	This study
*CYC8-3×FLAG (BTY234)*	*MATa his3D1 leu2D0 met15D0 ura3D0 CYC8–3×FLAG (kanR*)	This study
*dbp2*Δ* CYC8–3×FLAG (BTY248)*	*MATa dbp2::HygB his3D1 leu2D0 met15D0 ura3D0 CYC8–3×FLAG (kanR*)	This study
*xrn1*Δ* CYC8–3×FLAG (BTY249)*	*MATa xrn1::HygB his3D1 leu2D0 met15D0 ura3D0 CYC8–3×FLAG (kanR)*	This study

All strains in the BY4741 background unless otherwise noted.

**Table 3:** Oligos for strain construction.

DBP2 KO F	CAACAACCTGTAACAGAATTAAGCACTATTAAGGCAAATTTAGAGCAAA TATGCAGCTGAAGCTTCGTACGC
DBP2 KO R	GCAGTCAACTTATATAATTATTATTAATAGAGATGAATGAATTGAATCA CTTTGGCATAGGCGACTAGTGGATCTG
XRN1 KO F	ATGGGTATTCCAAAATTTTTCAGGTACATCTCAGAAAGATGGCCCATG ATTTTACAGCTTTGCAGCTGAAGCTTCGTACGC
XRN1 KO R	CTAAGTAGATTCGTCTTTTTTATTATCACGGTCAGCAGCATTGCTTTGT GACTTTGGCGAGCATAGGCGACTAGTGGATCTG
DCP2 KO F	ATAATATTGCTTTGAATCTGAAAAAAATAAAAGTACCTTCGCATT AGACAATGCAGCTGAAGCTTCGTACGC
DCP2 KO R	GGCTGCCTTCATTTACAGTGTGTCTATAAAACGTATAACACTTATT CTTTGCATAGGCGACTAGTGGATCTG
CYC8-3×FLAG F	TGTAGTAAGGCAAGTGGAAGAAGATGAAAACTACGACGACAGGGA ACAAAAGCTGGAG
CYC8-3×FLAG R	GATTATAAATTAGTAGATTAATTTTTTGAATGCAAACTTTCTATAGGGCGAATTGGGT

### GAL Induction Analyses

Time courses were performed by growing strains at 30°C to an OD_600_ of 0.4 in YP 2% glucose or raffinose when indicated and shifting to 2% galactose media. 3OD units were harvested at 30 min time points from 0–180 min. Kinetic studies were conducted over a 300 min induction with 30 min time points with the inclusion of additional 10 min time points for higher resolution analysis of wild-type cells where indicated. Lag times, rates, and half times were calculated following autoradiography and quantification of abundance with respect to the *SCR1* loading control and a *GAL* control RNA when indicated. The *GAL* control RNA corresponds to RNA isolated from an isogenic wild-type strain following a 300 min induction from raffinose and is utilized as a control for full induction. Estimated lag times are independent of final, steady-state levels and correspond to the first time point in a series with increasing *GAL* mRNA signal above background after normalization to *SCR1*. Lag time error between biological replicates is reported as the standard deviation to illustrate the range of variation. Transcript levels were determined as the percentage of a wild-type control using the following equation: (*GAL* Transcript Signal/*SCR1* signal)÷(*GAL* Control/*SCR1* Control)×100%, whereby *GAL* positive corresponds to total RNA from a wild-type culture following a 300-min induction from derepressive (+raffinose) conditions. Transcriptional profiles were fitted to a dose response curve with variable slope in GraphPad Prism using the following equation: Y = lowest level+(highest level−lowest level)÷(1+10∧((T_1/2_−X)×HillSlope)). T_max_ corresponds to the first time point in a series when the *GAL* mRNA signal reaches a steady-state plateau, whereas initial velocities were determined by calculating the slope of a straight line from the lag time to the T_max_. T_1/2_ times correspond to the average time to reach 50% maximum transcript levels within the cell population. Calculated lag times in Figure 7 were determined by fitting transcriptional induction data points for each biological replicate to a multivariable, exponential growth curve (DM Fit v. 2.0) [54] and are reported as the average with the s.d. All experiments were conducted with three biological replicates with error between transcript levels as SEM.

### RNA Isolation and Quantitation

RNA extraction, northern blotting, and RT-qPCR were performed as in [37]. Probes were made from PCR products using the DNA plasmid templates listed in Table 1. RT-qPCR primers are listed in Table 4. Primers for Northern blotting probes are listed in Table 5.

**Table 4:** RT-qPCR oligos.

nc10 F	GAGGTCTTGACCAAGCATCACA
nc10 R	TTCCAGACCTTTTCGGTCACA
nc7 F	TGAACAAGCCATATGGAGACA
nc7 R	CGACGATATTACCCGTAGGAA
GAL10 5′ F	GAGGTCTTGACCAAGCATCACA
GAL10 5′ R	TTCCAGACCTTTTCGGTCACA
GAL7 5′ F	CAAAAAGCGCTCGGACAACT
GAL7 5′ R	GCTTGGCTATTTTGTGAACACTGT
ACT1 F	TGGATTCCGGTGATGGTGTT
ACT1 R	TCAAAATGGCGTGAGGTAGAGA

**Table 5:** Oligos for northern blotting (dsDNA probes).

SCR1 F	GGATACGTTGAGAATTCTGGCCGAGG
SCR1 R	AATGTGCGAGTAAATCCTGATGGCACC
GAL7 F	CCTTGGTTAGGTCAACAGGAG
GAL7 R	AGTCGCATTCAAAGGAGCC
GAL10 F	GCATCACATTCCCTTCTATGAG
GAL10 R	ACGATTAGCATACCTGCCG
GAL1 F	TTGGACGGTTCTTATGTCAC
GAL1 R	GAGACTCGTTCATCAAGGC

### ChIP Analysis

ChIP was performed as described previously [37], with the following modifications. After formaldehyde fixation, cells were pelleted and washed twice with cold wash buffer (50 mM HEPES•KOH, 140 mM NaCl, and 1 mM EDTA) and frozen in liquid nitrogen. Cells were then lysed cryogenically using a Retsch Oscillating Mill MM400. Cell lysates were then resuspended in cold Lysis Buffer (50 mM HEPES•KOH, 140 mM NaCl, 1 mM EDTA, 1% Triton X-100, 0.1% sodium deoxycholate, 1 mM PMSF and 1× protease inhibitor (complete, ETDA-free, Roche)), and chromatin was sheared by sonication. For Cyc8–3×flag ChIP, chromatin from ∼1.4×10^8^ cells was immunoprecipitated with 1 µL of FLAG M2 monoclonal antibody (F3165, Sigma) and 12 µL of Protein G Dynabeads (30 mg/mL, Invitrogen) at 4°C for 2 h. For PolII ChIP, chromatin from 2–3×10^8^ cells was immunoprecipitated with 1 µL of Rpb3 monoclonal antibody (WP012, Neoclone) and 12 µL of Protein G Dynabeads (30 mg/mL, Invitrogen) at 4°C for 2 h. Immunoprecipitated DNA was isolated as described previously [37]. Quantitative PCR was performed using Bio-Rad CFX96 Real-time system using PrimeTime Assay primers purchased from IDT (Table 6). All ChIP experiments were performed with three biological replicates and three technical repeats. Error bars represent the SEM of three biological replicates.

**Table 6:** Primetime assays for ChIP.

Name	Forward	Reverse	Probe
GAL10 promoter	CTTTATTGTTCGGAGCAGTGC	GCTCATTGCTATATTGAAGTACGG	CGGTGAAGACGAGGACGCACG
GAL10 5′	TGGTGCTGGATACATTGGTTC	AGGGAATGTGATGCTTGGTC	TGACTGTGTTGTTGCTGATAACCTGTCG
GAL7 promoter	GCGCTCGGACAACTGTTG	TTTCCGACCTGCTTTTATATCTTTG	CCGTGATCCGAAGGACTGGCTATACA
GAL7 5′	ATCATACAATGGAGCTGTGGG	CTAGCCATTCCCATAGACGTTAC	AAGCAGCCTCCTGTTGACCTAACC
GAL6 promoter	CCAGAAAGTCACCTGCTCTC	GCATGTAACAAAAGAGCAAGGG	CGCCGACGGGCACCCATAA
ACT1 middle	ATTGAGAGTTGCCCCAGAAG	ATGGAAACGTAGAAGGCTGG	ACACCC TGTTCTTTTGACTGAAGCTCC

### Yeast Cell Lysate Preparation and Western Blotting

Yeast cells were grown in YP 2% glucose to an O.D. of 0.4–0.6. We harvested 30 mg of yeast cells and lysed them with 1.85 M NaOH and 7.4% β-mercaptoethanol on ice for 10 min. Proteins were precipitated with 50% TCA on ice for 10 min and resuspended into 300 µl 1×SDS-PAGE loading dye. We then resolved 1–1.5 mg proteins by SDS-PAGE and transferred them to a nitrocellulose membrane. FLAG-tagged Cyc8 and Pgk1 were detected by rabbit anti-3×FLAG (F7425, Sigma) and monoclonal mouse anti-yeast Pgk1 (459250, Invitrogen), respectively. Proteins were visualized by alkaline phosphatase-based detection using AP-conjugated anti-rabbit secondary antibody and AP-conjugated anti-mouse secondary antibody, respectively, followed by a BCIP/NBT chemistry (S3771, Promega).

## Supporting Information

Figure S1 **Representative northern blots for** ***GAL7*** **and** ***GAL10*** **induction from derepressed conditions in** ***XRN1*** **-deficient cells.** (A–B) *GAL7* (A) and *GAL10* (B) induction profile of one biological replicate for wild -type, *lncRNA*Δ*, xrn1*Δ*, and xrn1*Δ* lncRNA*Δ* strains from derepressed conditions. Transcriptional induction assays were conducted from cells grown in derepressive (+raffinose) to activated (+galactose) conditions. GAL7 and GAL10 transcripts were detected by northern blotting using a ^32^P-labeled double-stranded DNA probe as in Figure 1. SCR1 was detected similarly and serves as a loading control. Lag times correspond to the average time to detection of GAL transcripts for the three independent biological replicates shown in Figure 5 following normalization to SCR1 and the control RNA (not pictured). Note that bands are detectible in wild-type and lncRNAΔ strains in (B) at the 30 min time point (yielding similar lag times for all strains), but appear weaker than in xrn1Δ strains due to loading differences between blots.*
(TIF)

Figure S2 **Representative northern blots for** ***GAL7*** **and** ***GAL10*** **induction from repressed conditions in** ***XRN1*** **-deficent cells.** (A–B) *GAL7* (A) and *GAL10* (B) induction profile of one biological replicate for wild-type, *lncRNA*Δ*, xrn1*Δ*, and xrn1*Δ* lncRNA*Δ* strains from repressed conditions. Transcriptional induction assays were conducted as above during the switch from repressed (+glucose) to activated (+galactose) conditions. Lag times correspond to the average time to detection of GAL transcripts for the three, independent biological replicates shown in Figure 6 and are calculated following normalization to SCR1 and the GAL control.*
(TIF)

Figure S3 **Transcriptional induction assays for wild-type and** ***lncRNA***Δ** strains from repressed to activated conditions.** (A–B) High-resolution analysis of transcriptional induction in wild-type and *lncRNA*Δ* cells. Transcription induction was measured in wild-type or lncRNAΔ cells from repressed conditions as above with the inclusion of additional 10 min time points from 90–150 min immediately prior to recruitment of RNAPII (see Figure 4). Lag times are not determined visually from the blots but were calculated as the average across three biological replicates after normalization to the SCR1 loading control.*
(TIF)

Figure S4 **Individual transcriptional induction profiles following curve fitting analysis.** Individual biological replicates of induction profiles of wild-type and *lncRNA*Δ* strains from repressed to activated conditions. Transcript levels were normalized to SCR1 and the GAL “control” RNA as above. Resulting data points were then fit to a dynamic exponential growth curve (DM fit v. 2.0) [54]. R^2^ values and lag times are shown for each individual profile. Calculated lag times are reported in Figure 7 (C and D) and correspond to the average lag time and s.d. for induction of GAL7 and GAL10 after curve fitting for wild-type and lncRNAΔ strains.*
(TIF)

## Abbreviations

ChIP: chromatin immunoprecipitation
*GAL*: galactose metabolic gene
lncRNA: long noncoding RNA
mRNA: messenger RNA
RT-qPCR: reverse transcriptase-quantitative PCR
Vi: initial velocity
