# Supplementary figures and images for: Long Noncoding RNAs Promote Transcriptional Poising of Inducible Genes
Source: PLoS Biol. 2013 Nov 19;11(11):e1001715. doi: 10.1371/journal.pbio.1001715 (PMC3833879; doi:10.1371/journal.pbio.1001715)

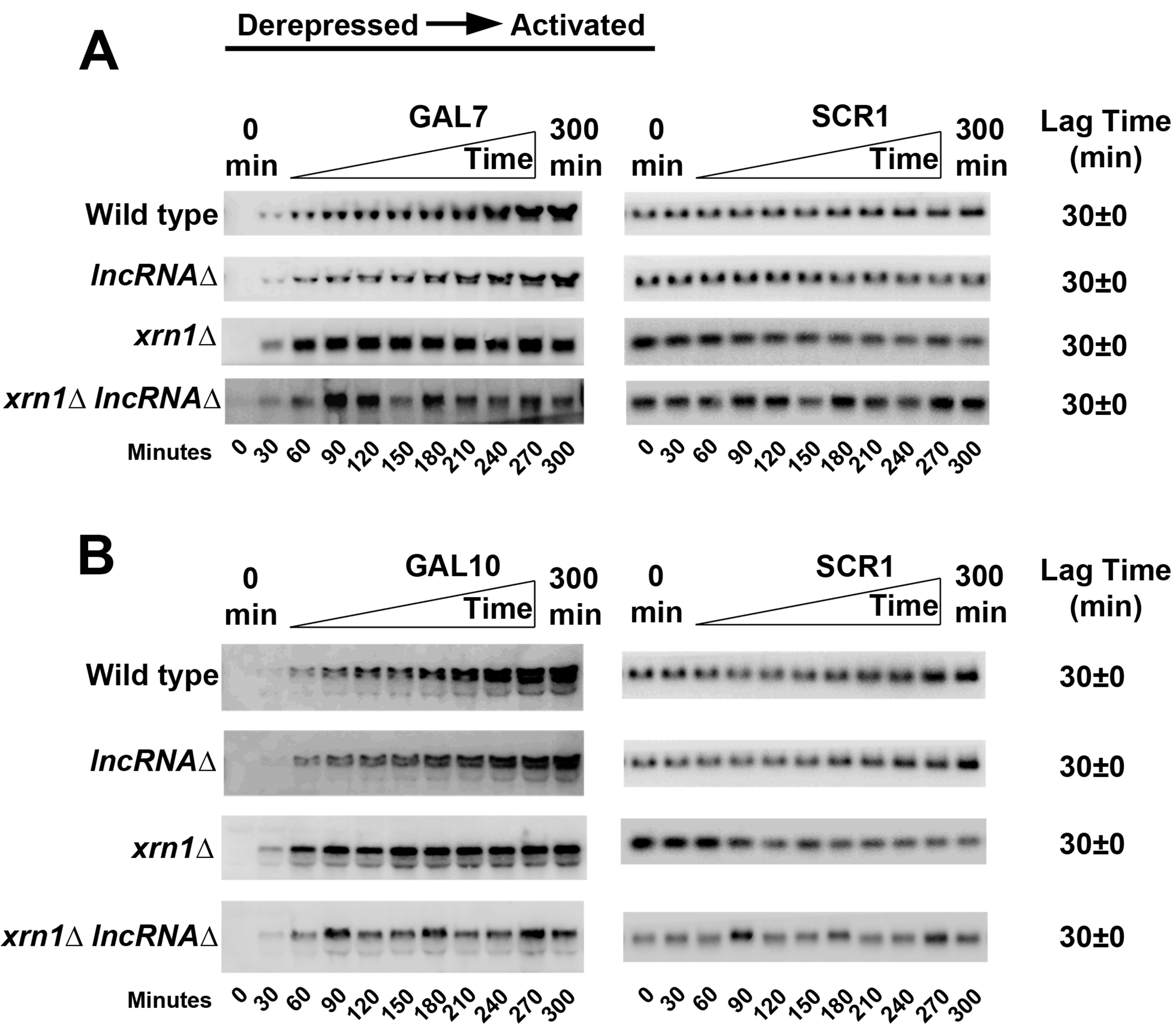

Supplement: Figure S1 — Representative northern blots for GAL7 and GAL10 induction from derepressed conditions in XRN1 -deficient cells. (A–B) GAL7 (A) and GAL10 (B) induction profile of one biological replicate for wild -type, lncRNAΔ, xrn1Δ, and xrn1Δ lncRNAΔ strains from derepressed conditions. Transcriptional induction assays were conducted from cells grown in derepressive (+raffinose) to activated (+galactose) conditions. GAL7 and GAL10 transcripts were detected by northern blotting using a 32P-labeled double-stranded DNA probe as in Figure 1. SCR1 was detected similarly and serves as a loading control. Lag times correspond to the average time to detection of GAL transcripts for the three independent biological replicates shown in Figure 5 following normalization to SCR1 and the control RNA (not pictured). Note that bands are detectible in wild-type and lncRNAΔ strains in (B) at the 30 min time point (yielding similar lag times for all strains), but appear weaker than in xrn1Δ strains due to loading differences between blots. (TIF) [file pbio.1001715.s001.tif]

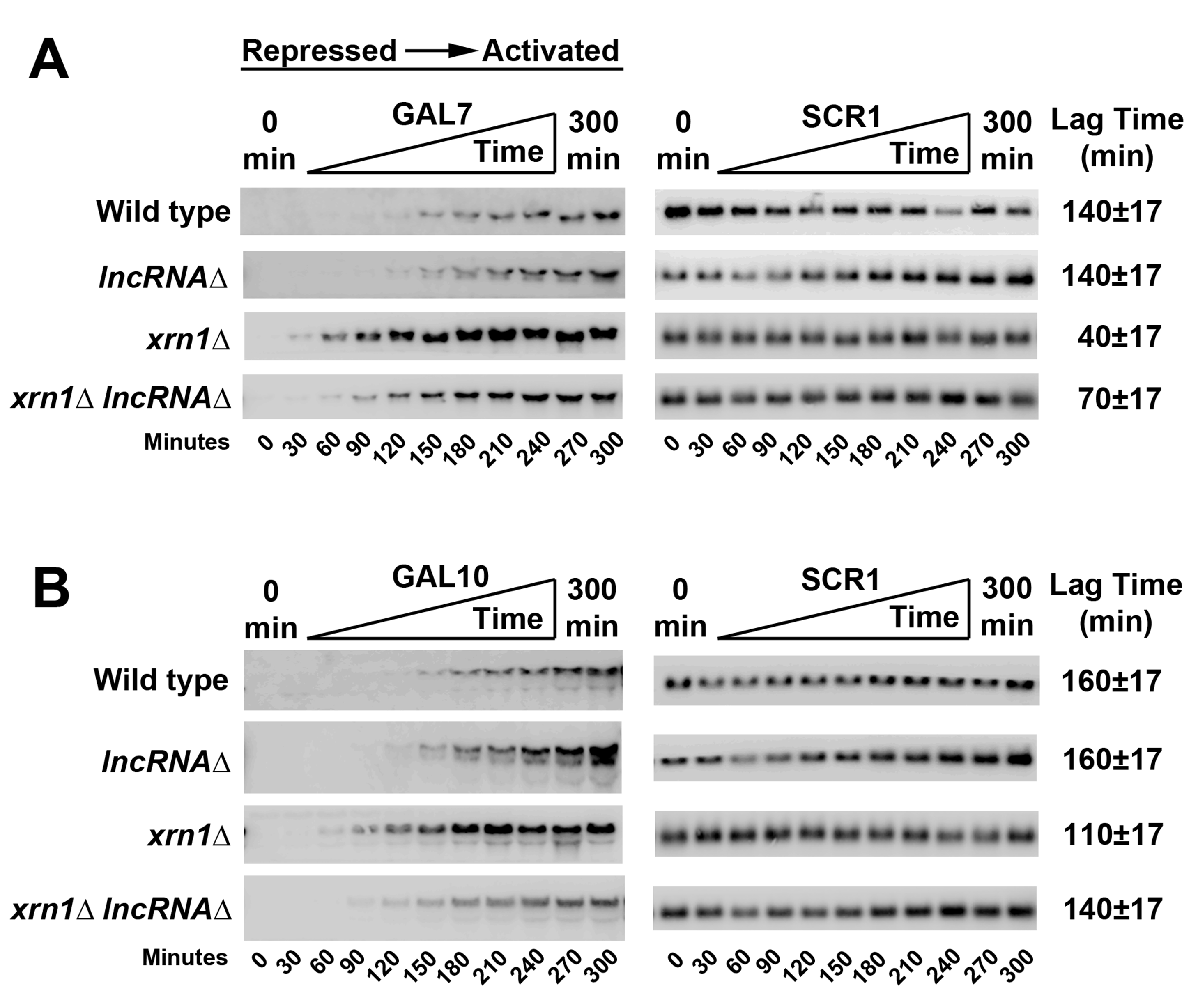

Supplement: Figure S2 — Representative northern blots for GAL7 and GAL10 induction from repressed conditions in XRN1 -deficent cells. (A–B) GAL7 (A) and GAL10 (B) induction profile of one biological replicate for wild-type, lncRNAΔ, xrn1Δ, and xrn1Δ lncRNAΔ strains from repressed conditions. Transcriptional induction assays were conducted as above during the switch from repressed (+glucose) to activated (+galactose) conditions. Lag times correspond to the average time to detection of GAL transcripts for the three, independent biological replicates shown in Figure 6 and are calculated following normalization to SCR1 and the GAL control. (TIF) [file pbio.1001715.s002.tif]

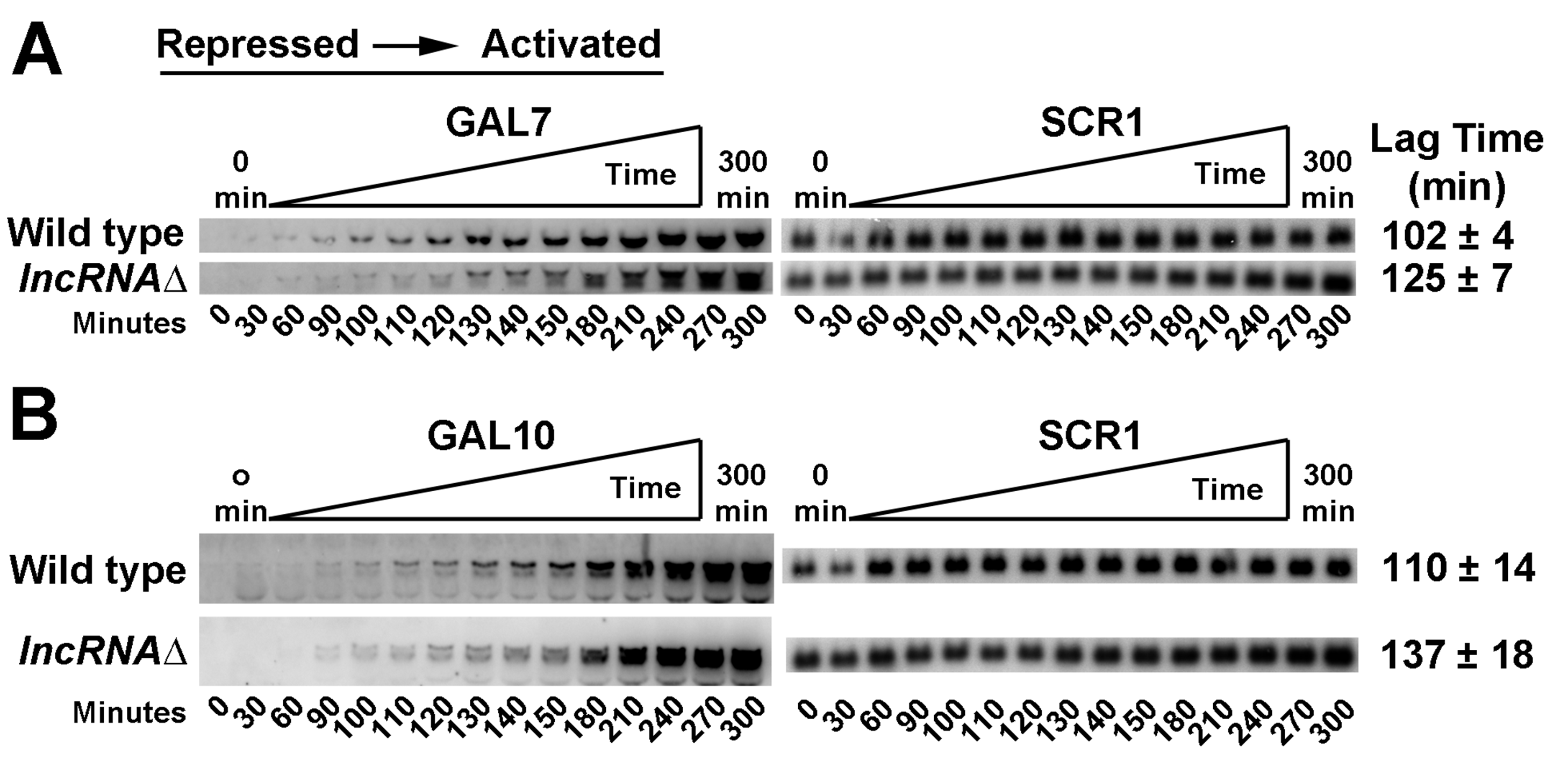

Supplement: Figure S3 — Transcriptional induction assays for wild-type and lncRNAΔ strains from repressed to activated conditions. (A–B) High-resolution analysis of transcriptional induction in wild-type and lncRNAΔ cells. Transcription induction was measured in wild-type or lncRNAΔ cells from repressed conditions as above with the inclusion of additional 10 min time points from 90–150 min immediately prior to recruitment of RNAPII (see Figure 4). Lag times are not determined visually from the blots but were calculated as the average across three biological replicates after normalization to the SCR1 loading control. (TIF) [file pbio.1001715.s003.tif]

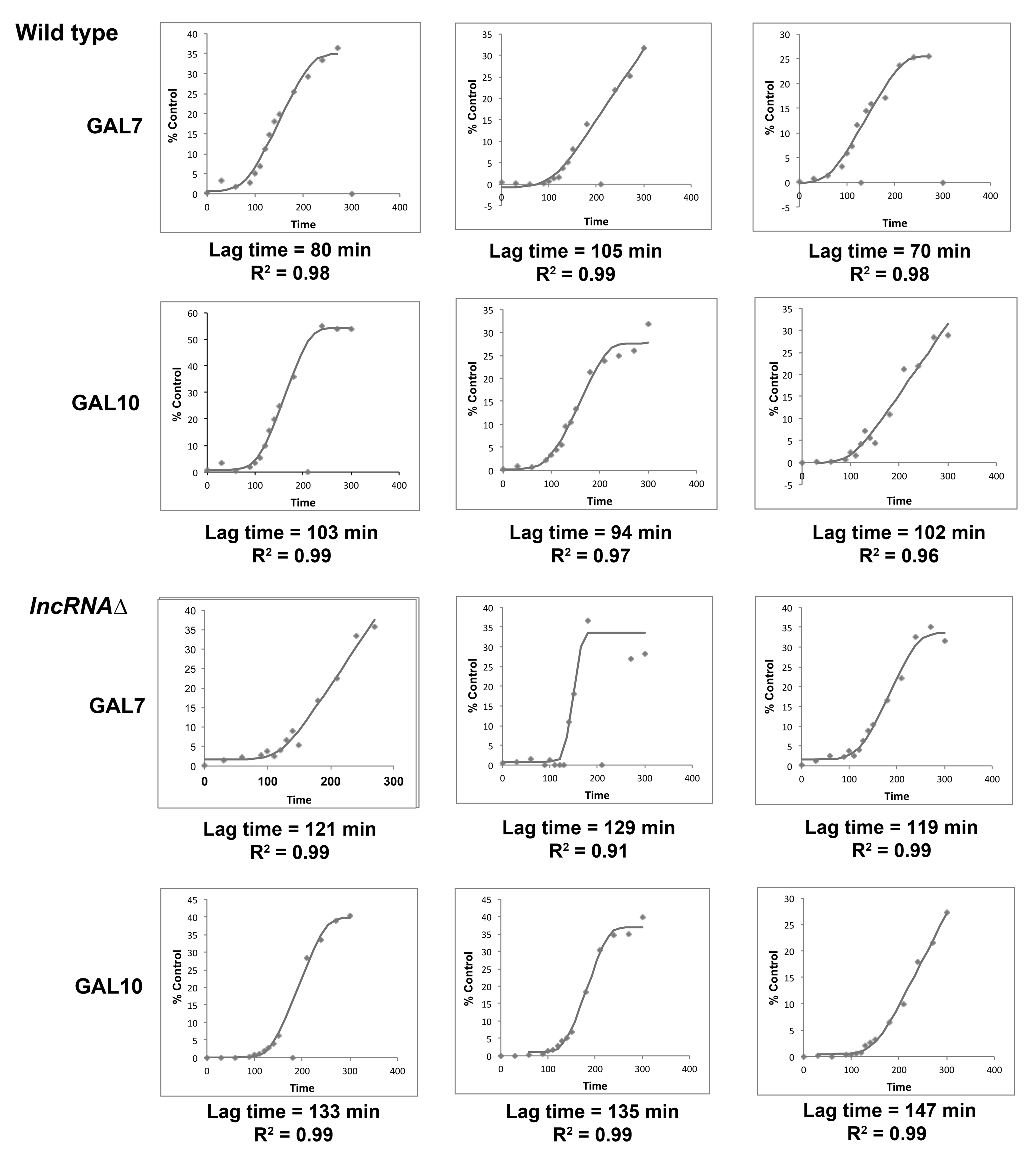

Supplement: Figure S4 — Individual transcriptional induction profiles following curve fitting analysis. Individual biological replicates of induction profiles of wild-type and lncRNAΔ strains from repressed to activated conditions. Transcript levels were normalized to SCR1 and the GAL “control” RNA as above. Resulting data points were then fit to a dynamic exponential growth curve (DM fit v. 2.0) [54]. R2 values and lag times are shown for each individual profile. Calculated lag times are reported in Figure 7 (C and D) and correspond to the average lag time and s.d. for induction of GAL7 and GAL10 after curve fitting for wild-type and lncRNAΔ strains. (TIF) [file pbio.1001715.s004.tif]
